# Trends in Number and Distribution of COVID-19 Hotspot Counties — United States, March 8–July 15, 2020

**DOI:** 10.15585/mmwr.mm6933e2

**Published:** 2020-08-21

**Authors:** Alexandra M. Oster, Gloria J. Kang, Amy E. Cha, Vladislav Beresovsky, Charles E. Rose, Gabriel Rainisch, Laura Porter, Eduardo E. Valverde, Elisha B. Peterson, Anne K. Driscoll, Tina Norris, Nana Wilson, Matthew Ritchey, Henry T. Walke, Dale A. Rose, Nadia L. Oussayef, Monica E. Parise, Zack S. Moore, Aaron T. Fleischauer, Margaret A. Honein, Emilio Dirlikov, Julie Villanueva

**Affiliations:** ^1^CDC COVID-19 Response Team; ^2^Applied Physics Laboratory, Johns Hopkins University, Laurel, Maryland; ^3^Division of Public Health, North Carolina Department of Health and Human Services, Raleigh, North Carolina.

The geographic areas in the United States most affected by the coronavirus disease 2019 (COVID-19) pandemic have changed over time. On May 7, 2020, CDC, with other federal agencies, began identifying counties with increasing COVID-19 incidence (hotspots) to better understand transmission dynamics and offer targeted support to health departments in affected communities. Data for January 22–July 15, 2020, were analyzed retrospectively (January 22–May 6) and prospectively (May 7–July 15) to detect hotspot counties. No counties met hotspot criteria during January 22–March 7, 2020. During March 8–July 15, 2020, 818 counties met hotspot criteria for ≥1 day; these counties included 80% of the U.S. population. The daily number of counties meeting hotspot criteria peaked in early April, decreased and stabilized during mid-April–early June, then increased again during late June–early July. The percentage of counties in the South and West Census regions* meeting hotspot criteria increased from 10% and 13%, respectively, during March–April to 28% and 22%, respectively, during June–July. Identification of community transmission as a contributing factor increased over time, whereas identification of outbreaks in long-term care facilities, food processing facilities, correctional facilities, or other workplaces as contributing factors decreased. Identification of hotspot counties and understanding how they change over time can help prioritize and target implementation of U.S. public health response activities.

Aggregate, cumulative counts of reported COVID-19 cases (*1*) were collected by USAFacts through automated extraction or manual entry of information from state and local health department websites.^†^ CDC and the Applied Physics Laboratory (APL) of Johns Hopkins University cleaned the data to ensure nonnegative daily case counts and correct reporting errors (such as instances of 2 days’ of data being recorded on a single day) and analyzed data by county and report date. Hotspot counties were identified among counties in U.S. states and the District of Columbia by applying standardized criteria developed through a collaborative process involving multiple federal agencies; hotspots were defined based on relative temporal increases in number of cases.^§^ Prospective hotspot detection began on May 7, 2020. The same methods were applied retrospectively to detect hotspot counties using data from January 22, when the first U.S. COVID-19 case was reported (*2*), until May 6, 2020; no counties met hotspot criteria during January 22–March 7, 2020. Data from prospective and retrospective hotspot detection were analyzed to characterize trends in COVID-19 hotspot counties and hotspot alerts (each time a county meets hotspot criteria for 1 day) over time. Counties meeting hotspot criteria were analyzed by U.S. Census region (*3*) and urbanicity^¶^ (*4*).

CDC and APL assessed factors contributing to increased COVID-19 cases in hotspot counties identified during May 11–July 13 by reviewing case and laboratory data from HHS Protect (https://protect-public.hhs.gov/), a secure data hub for sharing COVID-19 information for first responders, researchers, and policy-makers; health department websites; online news reports; CDC deployment information; and outreach to state health department leadership to validate the contributing factors. A county could have more than one contributing factor identified. Contributing factors included focal outbreaks (i.e., at long-term care facilities, food processing facilities, correctional facilities, or other workplaces), community transmission, increased testing or irregular reporting, or no discernible cause. Analysis identified differences in contributing factors, comparing community transmission versus focal outbreaks, for counties identified in May compared with those identified in June and July.

During March 8–July 15, 2020, among the 3,142 U.S. counties, 818 (26%) met hotspot criteria for ≥1 day for a total of 9,898 alerts (Figure 1). These 818 counties include 80% of the U.S. population. The median number of days (not necessarily consecutive) that a county met the hotspot criteria during March 8–July 15 was 10 (interquartile range = 5–18). The daily number of counties meeting hotspot criteria peaked at 175 in early April, decreased and stabilized at <75 per day during mid-April to early June, then increased again to 179 in early July (Figure 2).

**FIGURE 1:**
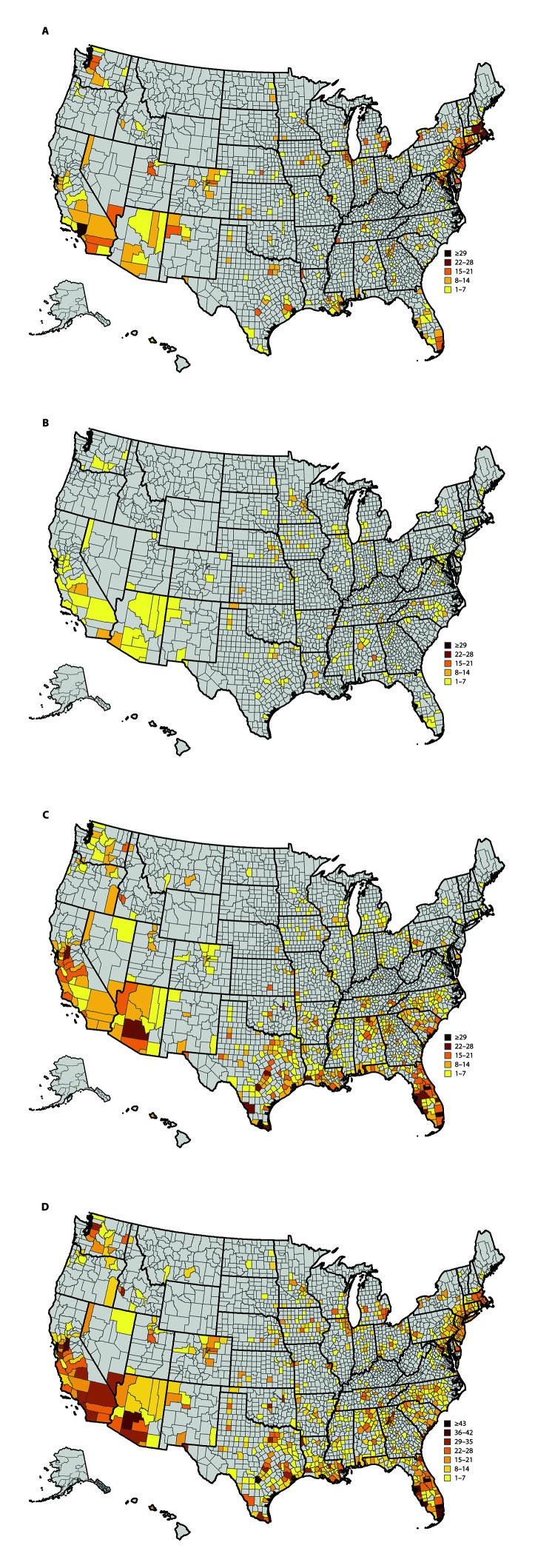
Number of COVID-19 hotspot alerts, by county and number of days* meeting hotspot criteria for (A) March 8–April 30, (B) May 1–31, (C) June 1–July 15, and (D) entire period — United States, March 8–July 15, 2020 **Abbreviation:** COVID-19 = coronavirus disease 2019. * Each county is shaded according to the number of days that the county met hotspot criteria, with shading in 7-day increments.

**FIGURE 2:**
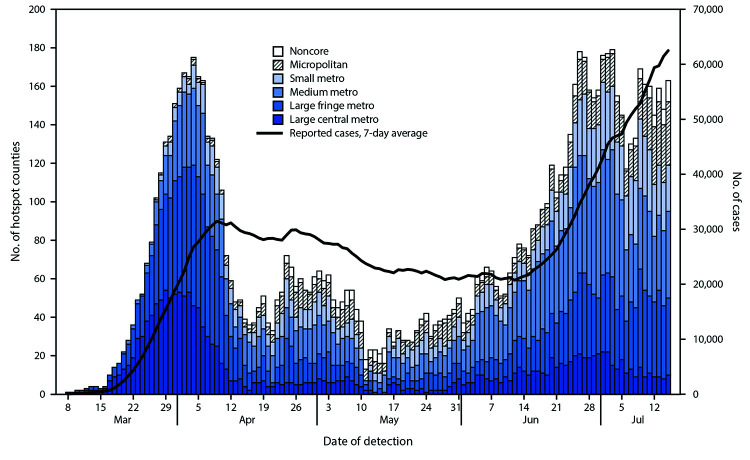
Daily number of COVID-19 hotspot alerts, by urbanicity,* and 7-day average of new reported cases — United States, March 8–July 15, 2020^†^ **Abbreviation:** COVID-19 = coronavirus disease 2019. * According to CDC’s National Center for Health Statistics urban-rural classification scheme for counties. *Large central metro counties*: in metropolitan statistical areas (MSAs) of ≥1 million population that contain all or part of the area's principal city. *Large fringe metro counties*: in MSAs of ≥1 million population and do not qualify as large central. *Medium metro counties*: in MSAs of 250,000–999,999 population. *Small metro counties*: in MSAs of <250,000 population. *Micropolitan counties*: in micropolitan statistical area. *Noncore counties*: not in metropolitan or micropolitan statistical areas. ^†^ No hotspots were detected during January 22–March 7, 2020.

By U.S. Census region, the percentage of counties meeting hotspot criteria differed over time (Table). During March–April, 40% of northeastern counties, representing 84% of the population of the Northeast region, met hotspot criteria for ≥1 day, whereas hotspot criteria were met by 8%–13% of counties in other regions. During May, 8%–11% of counties in all four U.S. Census regions met hotspot criteria. During June and July, 28% of southern counties, representing 76% of the population in the South Census region, and 22% of western counties, representing 86% of the population in the West Census region, met hotspot criteria, whereas 9%–10% of counties in the Northeast and Midwest, representing 16%–44% of the population in those regions, met hotspot criteria.

**TABLE:** Number of COVID-19 hotspot counties, by U.S. Census region* and urbanicity^†^ — United States, March 8–July 15, 2020^§^

Characteristic	No. (column %)		Unique hotspot counties^¶^							
			Total		March–April		May		June–July	
	Total U.S. counties	Total U.S. population	No. (row %) of counties	Row % of population	No. (row %) of counties	Row % of population	No. (row %) of counties	Row % of population	No. (row %) of counties	Row % of population
**Total**	**3,142 (100)**	**328,239,523 (100)**	**818 (26)**	**80**	**365 (12)**	**64**	**274 (9)**	**29**	**621 (20)**	**61**
U.S. Census region*										
Northeast	217 (7)	55,982,803 (17)	94 (43)	86	86 (40)	84	24 (11)	10	20 (9)	16
South	1,422 (45)	125,580,448 (38)	456 (32)	81	137 (10)	54	127 (9)	32	399 (28)	76
Midwest	1,055 (34)	68,329,004 (21)	163 (16)	67	84 (8)	52	83 (8)	32	104 (10)	44
West	448 (14)	78,347,268 (24)	105 (23)	86	58 (13)	75	40 (9)	36	98 (22)	86
**Urbanicity^†^**										
Large central metro	68 (2)	101,005,069 (31)	68 (100)	100	66 (97)	99	31 (46)	36	53 (78)	79
Large fringe metro	368 (12)	82,475,531 (25)	207 (56)	90	115 (31)	72	59 (16)	28	144 (39)	59
Medium metro	372 (12)	68,841,839 (21)	198 (53)	86	96 (26)	59	72 (19)	39	170 (46)	73
Small metro	358 (11)	29,854,023 (9)	140 (39)	63	47 (13)	22	46 (13)	22	116 (32)	53
Micropolitan	641 (20)	27,294,422 (8)	143 (22)	28	29 (5)	6	40 (6)	7	101 (16)	22
Noncore	1,335 (42)	18,768,639 (6)	62 (5)	9	12 (0)	2	26 (2)	4	37 (3)	6

**Abbreviation:** COVID-19 = coronavirus disease 2019.

* *Northeast*: Connecticut, Maine, Massachusetts, New Hampshire, New Jersey, New York, Pennsylvania, Rhode Island, and Vermont. *Midwest*: Illinois, Indiana, Iowa, Kansas, Michigan, Minnesota, Missouri, Nebraska, North Dakota, Ohio, South Dakota, and Wisconsin. *South*: Alabama, Arkansas, Delaware, District of Columbia, Florida, Georgia, Kentucky, Louisiana, Maryland, Mississippi, North Carolina, Oklahoma, South Carolina, Tennessee, Texas, Virginia, and West Virginia. *West*: Alaska, Arizona, California, Colorado, Hawaii, Idaho, Montana, Nevada, New Mexico, Oregon, Utah, Washington, and Wyoming.

^†^ According to CDC’s National Center for Health Statistics urban-rural classification scheme for counties. *Large central metro counties*: in metropolitan statistical areas (MSAs) of ≥1 million population that contain all or part of the area's principal city. Large fringe metro *counties*: in MSAs of ≥1 million population and do not qualify as large central. *Medium metro counties:* in MSAs of 250,000–999,999 population. *Small metro counties:* in MSAs of <250,000 population. *Micropolitan counties:* in micropolitan statistical area. *Noncore counties:* not in metropolitan or micropolitan statistical areas.

^§^ Hotspot counties ascertained retrospectively for March 8–May 6, 2020, and prospectively for May 7–July 15, 2020.

^¶^ Each county with at least one alert during the period is included.

The percentage of counties meeting hotspot criteria also varied over time by counties’ urbanicity. The percentage of large central metropolitan counties meeting hotspot criteria was 97% during March–April, 46% in May, and 78% during June–July; the proportions were lower for large fringe metropolitan counties (31%, 16%, and 39%, respectively). The proportion of counties in medium metropolitan areas meeting hotspot criteria during June–July was higher (46%) than the percentage during March–April (26%), as was true for counties in small metropolitan areas (32% versus 13%) and micropolitan areas (16% versus 5%). Few counties in noncore areas met hotspot criteria (1%–3%).

Factors contributing to increases in cases were identified for 116 (94%) of 124 counties with new hotspot alerts during May 11–31 (mean = 1.7 per county, total = 214), and for 389 (72%) of 539 counties with new alerts during June 1–July 13, (mean = 1.2 per county, total = 481). The proportion of factors contributing to the increases in reported COVID-19 cases that were focal outbreaks decreased from 56% during May 11–31 to 24% during June 1–July 13, whereas the proportion of identified factors that were community transmission increased from 18% to 41%, and the proportion not related to any discernible factor increased from 8% to 24%. The proportion with increased testing or reporting delay identified as contributing factors decreased from 17% to 11%.

During May 7–July 15, CDC deployed 92 teams comprising 375 persons to 37 states and the District of Columbia; the majority of these deployments were related to hotspots. For example, in response to requests for assistance with hotspot counties, CDC and the U.S. Public Health Service (USPHS; https://www.usphs.gov/) deployed multidisciplinary teams to North Carolina beginning June 13 (CDC) and June 22 (USPHS) to assist with case investigation, contact tracing, and data management; the CDC Foundation (https://www.cdcfoundation.org/) provided additional contact tracers to support local health departments in North Carolina in managing these hotspots. These public health staff members collaborated with the extensive network of local and state health officials responding to the pandemic.

## Discussion

Identifying hotspot counties experiencing localized increases in COVID-19 incidence provides CDC and other federal, state, and local agencies critical information for understanding the changing epidemiology of COVID-19 and targeting the implementation of rapid public health response activities. After hotspot counties are identified, quantitative and qualitative data from multiple data sources (describing not only local epidemiology, but also demographic characteristics, prevention efforts, testing, and health care utilization) are used to inform outreach to local officials. Outreach to local officials provides an opportunity to validate findings, identify specific concerns within the community, and identify resources and opportunities for interventions adapted to the specific needs of the local area. Such intervention can include technical assistance from federal staff members upon request from state health departments, including deployments to support epidemiology and analysis, contact tracing, laboratory testing, community mitigation, worker safety, infection prevention and control, health communications, and health care. These types of partnerships, exemplified by the collaborative effort between CDC, North Carolina Department of Health and Human Services, USPHS, and the CDC Foundation, highlight the intensive local, state, and federal efforts being used across the country to focus urgent public health actions where they are needed most.

Increased community transmission during June and July demonstrated the speed with which SARS-CoV-2, the virus that causes COVID-19, can spread, even in the absence of outbreaks in high-risk congregate settings, such as long-term care facilities, food processing facilities, and correctional facilities (*5*,*6**,**7*). Increasing geographic spread across metropolitan and micropolitan counties with community transmission indicates a pressing need to strengthen community mitigation efforts, including use of face masks, physical distancing, and hand hygiene. Population characteristics as well as other cultural, language, and sociopolitical factors should be considered when developing and implementing locally adapted responses, ideally with engagement of local community leaders.

The findings in this report are subject to at least three limitations. First, identification of hotspot counties was based on aggregate data, and differences in testing availability, reporting delays, and changes in reporting over time might have affected the extent to which numbers of reported cases correlated with actual incidence. Second, in hotspot criteria, the absolute threshold for cases means that counties with smaller population sizes are less likely to be identified as a hotspot. Increases in cases are still monitored among smaller counties, with a focus on trends in neighboring counties. Finally, information on contributing factors was taken from data available from existing sources and might not have included all factors contributing to increased cases; availability of information might have varied for different communities.

Identification of hotspot counties permits a focused approach to assessment and response by local, state, and federal agencies. Efforts are underway to further improve methods to identify the most concerning hotspots, enabling enhanced response, and to detect communities at increased risk for becoming hotspots, facilitating earlier action. Rapid identification and characterization of hotspots will improve the timeliness and effectiveness of response efforts that can ultimately reduce the number of new COVID-19 cases.

**Summary**

**What is already known about this topic?**

U.S. geographic areas most affected by the COVID-19 pandemic have changed over time.

**What is added by this report?**

During March 8–July 15, 2020, 818 (26%) of 3,142 U.S. counties were identified as COVID-19 hotspots (counties meeting specified criteria relating to temporal increases in number of cases and incidence); these counties included 80% of the U.S. population. More hotspots were identified in the South and West during June–July.

**What are the implications for public health practice?**

Identification of hotspot counties allows for a focused approach for assessing localized COVID-19 outbreaks and implementing targeted public health response activities.

## Footnotes

* U.S. Census regions: *Northeast:* Connecticut, Maine, Massachusetts, New Hampshire, New Jersey, New York, Pennsylvania, Rhode Island, and Vermont. *Midwest*: Illinois, Indiana, Iowa, Kansas, Michigan, Minnesota, Missouri, Nebraska, North Dakota, Ohio, South Dakota, and Wisconsin. *South:* Alabama, Arkansas, Delaware, District of Columbia, Florida, Georgia, Kentucky, Louisiana, Maryland, Mississippi, North Carolina, Oklahoma, South Carolina, Tennessee, Texas, Virginia, and West Virginia. *West:* Alaska, Arizona, California, Colorado, Hawaii, Idaho, Montana, Nevada, New Mexico, Oregon, Utah, Washington, and Wyoming.

†
https://usafacts.org/articles/detailed-methodology-covid-19-data/ .

§ Counties defined as hotspot counties met all four of the following criteria, relative to the date assessed: 1) >100 new COVID-19 cases in the most recent 7 days, 2) an increase in the most recent 7-day COVID-19 incidence over the preceding 7-day incidence, 3) a decrease of <60% or an increase in the most recent 3-day COVID-19 incidence over the preceding 3-day incidence, and 4) the ratio of 7-day incidence/30-day incidence exceeds 0.31. In addition, hotspots must have met at least one of the following criteria: 1) >60% change in the most recent 3-day COVID-19 incidence, or 2) >60% change in the most recent 7-day incidence.

¶ According to the CDC’s National Center for Health Statistics urban-rural classification scheme for counties. *Large central metro counties*: in metropolitan statistical areas (MSAs) of ≥1 million population that contain all or part of the area's principal city. *Large fringe metro counties*: in MSAs of ≥1 million population and do not qualify as large central. *Medium metro counties*: in MSAs of 250,000–999,999 population. *Small metro counties*: in MSAs of <250,000 population. *Micropolitan counties*: in micropolitan statistical area. *Noncore counties*: not in metropolitan or micropolitan statistical areas.
